# PWDD-Net: A Patterned Wafer Defect Detection Network for Semiconductor Manufacturing

**DOI:** 10.3390/nano16161026

**Published:** 2026-08-19

**Authors:** Wenjie Kong, Wenyuan Zhang, Ling Qin, Dinghai Gong

**Affiliations:** 1 School of Artificial Intelligence, Guangxi Polytechnic University, Nanning 530226, China; kongwenjie1989@163.com (W.K.); wyzhang321@gmail.com (W.Z.); 2 Jiujiang Research Institute, Xiamen University, Xiamen 361005, China

**Keywords:** patterned wafer defect, deep learning, lightweight, semiconductor manufacturing

## Abstract

Various wafer defects appear inevitably, due to the highly complex and precise semiconductor fabrication processes. Thus, precise and rapid detection of patterned wafer surface defects is essential to prevent circuit failures and ensure product quality. Accordingly, a novel lightweight detection network termed PWDD-Net is proposed in this work, by introducing several modifications on YOLO11-nano. First, a novel LGE block, incorporating spatial and channel transformation with adaptive gated mechanism, is developed to enhance fine-grained feature extraction and representation. Second, by integrating a self-calibration block, a lightweight SC-C3k2 module is proposed to improve global feature capture while preserving network efficiency. Finally, the Slide loss is employed to distinguish easy and hard instances, thereby mitigating the imbalanced class distribution and improving classification precision. Experimental results show that PWDD-Net achieves a mAP@0.5 of 74.4% and a mAP@0.5:0.95 of 46.5%, yielding remarkable increments of 4.2% and 2.1% over the YOLO11-nano baseline, respectively. In addition, the network maintains a comparable parameter scale to the baseline and performs an inference speed of 78 FPS using an NVIDIA RTX 3080Ti GPU. These results demonstrate the model’s potential for real-time industrial wafer defect inspection applications.

## 1. Introduction

Semiconductor integrated circuits (ICs) are the core component of modern electronic information technology. They promote the rapid development of specific domains such as artificial intelligence, high-speed telecommunications and intelligent manufacturing [1]. The reliability of these electronic components is the prerequisite for robust performance of electronic products. As the main substrate for chip fabrication, silicon wafers always contain exponentially increasing transistor arrays and interconnection lines. The fabrication process involves many complex and precise cycles of photolithography, etching, and cleaning. Therefore, wafer quality inspection has become increasingly essential in the entire semiconductor manufacturing chain [2].

During the actual wafer production process, various surface defects on the wafer with circuit patterns inevitably arise due to uncontrollable factors, such as material impurity, thermal fluctuation, photolithography alignment deviation and equipment mechanical wear. The common defect types for patterned wafers include scratch, contamination, block etch, particle, uneven coating, and open/short circuit. As typical quality control targets in micro- and nanoscale semiconductor manufacturing, these defects generally exhibit dimensions ranging from sub-micron to micron scales, which has been widely recognized in the field of wafer manufacturing quality inspection [3,4,5]. Such small defects will damage the circuit layout on the wafer surface, resulting in chip failure. Therefore, accurate and rapid detection of these defects is critical for process optimization and quality assurance in semiconductor fabrication. Given the escalating density of interconnections, the identification of minute circuit anomalies on wafers has become increasingly difficult.

To address this challenge, various high-precision analytical techniques have been developed for nanoscale defect characterization of silicon microchips. Photoluminescence spectroscopy enables non-destructive analysis of crystal defects, impurity-related energy levels and carrier recombination behavior in silicon wafers. It is widely used for semiconductor material characterization, process monitoring, and quality assessment [6,7]. As a label-free nonlinear optical method, third-harmonic generation (THG) microscopy provides high-resolution three-dimensional (3D) imaging of subsurface interfaces and buried nanostructures, making it a powerful tool for defect inspection [8,9]. Electron and ion beam microscopy techniques are widely recognized as the gold standard for sub-nanometer defect characterization, enabling direct visualization of lattice-level defects as well as 3D tomographic analysis of microchip structures [10,11].

However, due to their high cost, low throughput and strict sample preparation requirements, these methods are primarily limited to offline laboratory-based failure analysis and are not suitable for high-throughput, full-wafer inline inspection in mass production environments.

Automated optical inspection (AOI) technology has been extensively deployed throughout the semiconductor manufacturing lines as a foundational tool for patterned wafer inspection. Initially, AOI systems used the comparative strategy, which segmented the wafer into discrete die units and performed differential analysis. Defects can be identified by comparing each die’s pattern against a defect-free reference template (die-to-reference) or adjacent dies (die-to-die). These comparative approaches are sensitive to sub-pixel registration errors and illumination variations [12]. Even sub-pixel misalignments or slight illumination changes can significantly decrease detection accuracy. In addition, traditional AOI heavily depends on hand-crafted features and template matching. Such conventional machine learning algorithms struggle to recognize tiny, low-contrast defects from dense and complex backgrounds. Moreover, these classical AOI methods require manual parameter tuning for every iteration due to their poor generalization ability.

In recent years, deep learning technologies, represented by convolution neural networks (CNNs) and vision Transformers, have provided considerable solutions for small object detection [13]. Thus, a variety of advanced deep learning networks have been proposed for inspecting wafer defects. For example, Rohan et al. [14] integrated a segmentation network and a Large Language Model (LLM) system to detect wafer map defects. Shinde et al. [15] investigated several You Only Look Once (YOLO) variants for wafer map defect detection, achieving 94% accuracy with a real-time inference. They also assessed other classical convolutional detectors such as ResNet [16] and DenseNet [17], both of which exhibited robust detection performance. Kim et al. [18] employed improved Xception [19] algorithm to inspect wafer buffer zones. The method performed accurate defect classification, precise localization, and reliable size determination, demonstrating its effectiveness for wafer inspection.

Despite the advancements in deep learning-based approaches, patterned wafer defect detection remains challenging. The complex circuits on the wafer surface introduce substantial background interference, which adversely affects discriminative feature extraction. Some defects occupy only a small region of the wafer, exhibiting weak characteristic signals, which are easily obstructed by the surrounding circuit patterns. Due to process variations in semiconductor manufacturing, the distribution of defects is always imbalanced. The class imbalance issue biases the learning process toward majority classes, thereby reducing the overall detection performance, especially for minority defects. In addition, many deep learning methods attempt to improve detection accuracy by increasing network depth or width. One common strategy is to deepen the network by stacking residual convolutional layers, thereby enhancing the learning capability of high-level semantic features, as widely demonstrated in classical backbone architectures [16,20]. Another approach involves widening the network by expanding feature channels and introducing multi-scale branch structures to enrich feature diversity [21,22]. However, such architecture expansion inevitably increases model complexity and computation cost, leading to decreased inference efficiency. Consequently, achieving an optimal balance between detection accuracy and inference speed is urgent.

To address the aforementioned limitations, we developed a novel patterned wafer defect detection network, named PWDD-Net, based on some improvements upon YOLO11-nano [23]. First, a lightweight gated extraction (LGE) block is newly designed to improve fine-grained feature extraction and enhance feature discrimination capability. The LGE enables adaptive capture of richer context information and high-resolution details. Second, a novel self-calibrated C3k2 (SC-C3k2) module is proposed to generate more discriminative feature maps, improving feature representation without increasing computation cost. Finally, Slide loss [24] is adopted to mitigate the imbalance issue between easy and hard samples through an adaptive weighting mechanism. As a result, the proposed PWDD-Net achieves rapid and accurate identification of patterned wafer surface defects with typical physical dimensions of sub-micron to tens of microns. The main contributions of this work can be summarized as follows:

1. Instead of conventional convolution, a novel LGE block is proposed to facilitate adaptive feature extraction and fusion. Group convolution is employed to efficiently map input features to the target dimensional space. Meanwhile, gated convolution is incorporated to dynamically strengthen informative features and suppress irrelevant features;
2. An SC-C3k2 module is designed by embedding a self-calibration block [25] into the original C3k2 structure. The self-calibration block enables the generation of a global receptive field without introducing additional parameters. Thus, the improved SC-C3k2 module produces more discriminative feature maps, improving the overall feature representation capability of the network;
3. Slide loss is introduced to replace the standard BCE loss to mitigate sample imbalance issue. By adopting an adaptive IoU-based threshold to distinguish easy and hard samples, it enhances boundary learning ability and improves generalization performance in imbalanced detection scenarios;

We review the relevant works in Section 2, and exhibit the architecture of PWDD-Net and clarify the proposed modifications in Section 3. Then, we illustrate and discuss the experimental results in Section 4, followed by a summary in Section 5.

## 2. Related Works

Semiconductor manufacturing consists of two major stages: wafer fabrication and integrated circuit (IC) production, corresponding to non-patterned and patterned wafer defects, respectively [26]. Multiple mechanical and chemical processes in wafer fabrication may introduce various structural and contamination-related defects, referred to as non-patterned wafer defects. In contrast, Photolithography realizes multilayer circuit pattern transfer through precise process steps [27]. Minor process variations may lead to pattern distortion, broken lines, particle contamination and other patterned defects. As lithography technology advances to 3–5 nm nodes, circuit patterns become more complicated and densely integrated [28]. Thus, the precise and efficient detection of patterned wafer defects is critically required.

The implementation of AOI techniques has optimized and upgraded the patterned wafer defect detection. Conventional AOI methods mainly adopted traditional image processing algorithms. For example, Shankar et al. [29] proposed a non-reference defect detection framework for wafer-die images. The model integrated discrete wavelet transform (DWT) with area sieves segmentation. This approach achieved superior defect segmentation performance compared to conventional wavelet-based reference methods. Tsai et al. [30] transformed gray solar wafer images into entropy maps of gradient directions, and employed mean shift smoothing to suppress heterogeneous grains. This method effectively detected surface fingerprints and contamination defects. Zhou et al. [31] combined common-path interferometric phase imaging with second-order difference filtering and matched-filter convolution to enable accurate detection of nanoscale defects.

Although these methods have demonstrated good performance, they largely depend on handcrafted features, image comparison or precise physical modeling. Such approaches always exhibit limited robustness and poor generalization under complex backgrounds. As circuit patterns become increasingly dense and complicated, traditional rule-based methods struggle to identify small defect features from strong interference. To address these limitations, deep learning-based methods have emerged for wafer defect detection. By automatically learning multi-level features, convolutional neural networks (CNNs) can provide improved adaptability to complex backgrounds and small defects.

Integrating a lightweight Transformer backbone, an adaptive hybrid encoder and an efficient residual fusion module, Mei et al. [26] proposed BDSD-Net to identify wafer surface defects efficiently. With the help of a knowledge distillation strategy, this method achieved competitive detection precision while maintaining high inference speed. Jou et al. [32] developed a CNN-based automated defect detection framework for ICs. It employed image augmentation techniques to supplement the scarce defect data, enhancing the model robustness. In contrast, to mitigate the high labeling cost, Ye et al. [33] introduced an unsupervised transformer-based model for wafer defect detection. This approach achieved favorable detection accuracy on both SEM and optical microscope images. Qiao et al. [34] proposed a residual attention based U-Net for fine-grained segmentation of semiconductor defects in SEM images. Benefiting from the improved feature extraction capability, the enhanced U-Net obtained an IoU of 71.92%.

Recently, YOLO series models have demonstrated excellent performance in industrial defect detection, including wafer defect inspection. Based on attention mechanism, adaptive feature representation and self-modulated feature fusion, Zhang et al. [35] proposed an enhanced YOLOv13 model for fast and accurate detection of wafer surface defects. Experimental results exhibited that the improved model outperformed the original YOLOv13, achieving 92.9% mAP@0.5. Similarly, Xu et al. [36] introduced a lightweight RFS-YOLOv8n to inspect patterned wafer defects. Their model enhanced feature representation ability and reduced model complexity, thereby balancing the detection accuracy and efficiency. The detection performance of 98.2% mAP@0.5 was achieved with a 34.7% reduction in parameter count.

In contrast to traditional image processing or conventional machine learning approaches, deep learning-based methods have achieved remarkable progress in patterned wafer surface defect detection. However, challenges such as class imbalance, fine-grained feature representation, large scale variance and computational efficiency remain critical issues. Therefore, it is imperative to develop a novel, lightweight, and accurate deep learning architecture for patterned wafer surface defect detection.

## 3. Materials and Methods

### 3.1. Data Preparation

#### 3.1.1. Data Acquisition

Patterned wafer surface defect images are limited and difficult to collect in large quantities from semiconductor manufacturing plants. Therefore, we utilized 4531 images from a public dataset [37], with an average resolution of 618 × 480 pixels. However, the original dataset was designed for segmentation tasks. In this study, to adapt them for object detection task, we annotated all images with bounding box labels using the X-AnyLabeling tool (CVHub520, Shenzhen, China) [38]. The resulting dataset comprises seven classes of wafer surface defects, including ‘block etch’, ‘coating bad’, ‘particle’, ‘PIQ particle’, ‘PO contamination’, ‘scratch’ and ‘SEZ burnt’.

**Figure 1:**
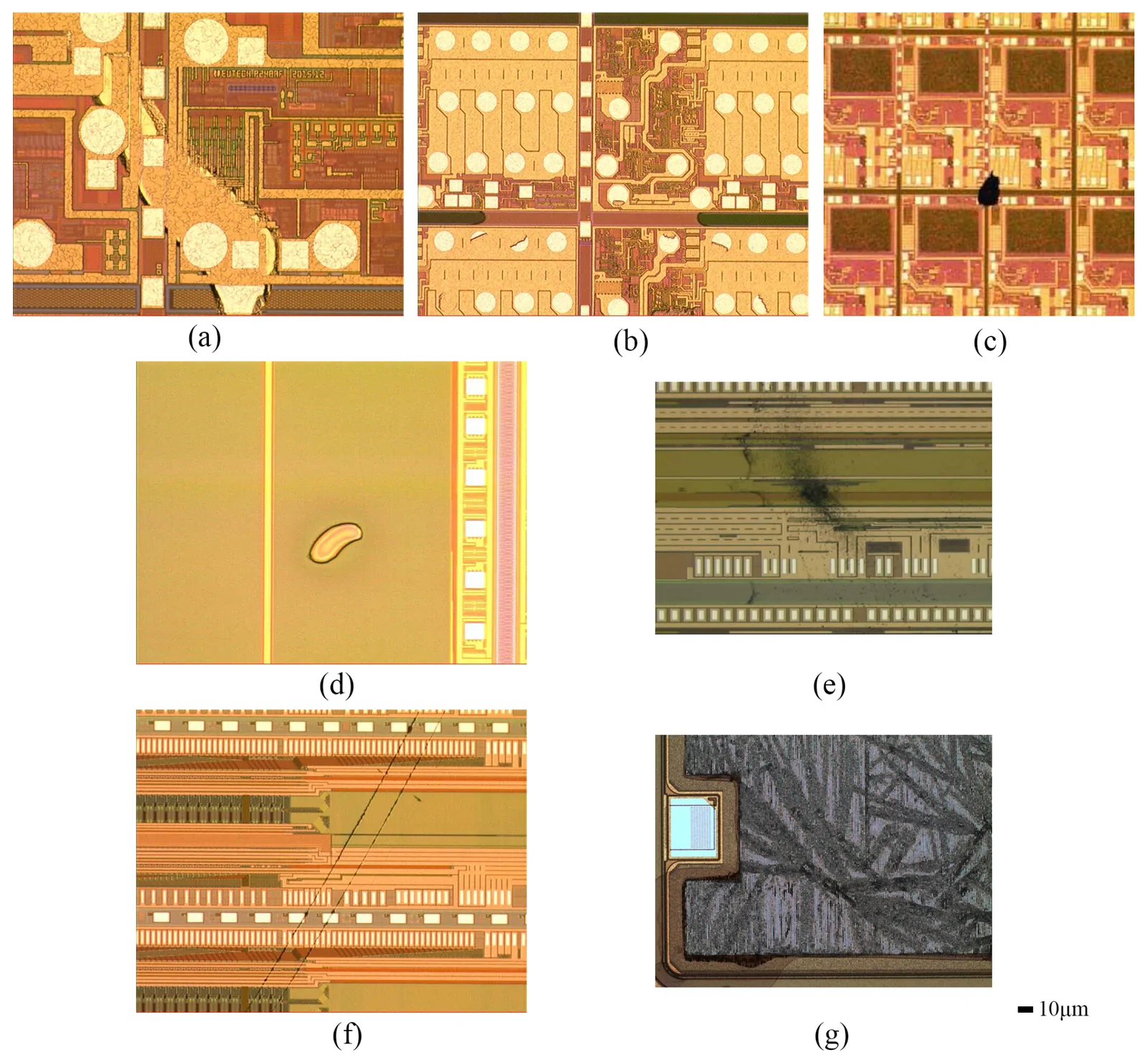
Wafer surface defect samples in the dataset: (**a**) block etch, (**b**) coating bad, (**c**) particle, (**d**) PIQ particle, (**e**) PO contamination, (**f**) scratch and (**g**) SEZ burnt. An estimated 10 μm scale bar is provided for dimensional reference.

The dataset was randomly split into training, validation and test subsets at a ratio of 7:2:1, comprising 3171, 906, and 454 images, respectively. To mitigate overfitting and enhance model generalization, the training subset was augmented using classical geometric transformations, such as rotation, flipping and transposition, resulting in a total of 12,684 training images. The detailed distribution of images among the three subsets and their input dimensions, are summarized in Table 1. Moreover, representative defect samples are illustrated in Figure 1. There are large scale variance among different defect categories and the strong background interference caused by complex patterned circuits.

#### 3.1.2. Data Analysis

**Figure 2:**
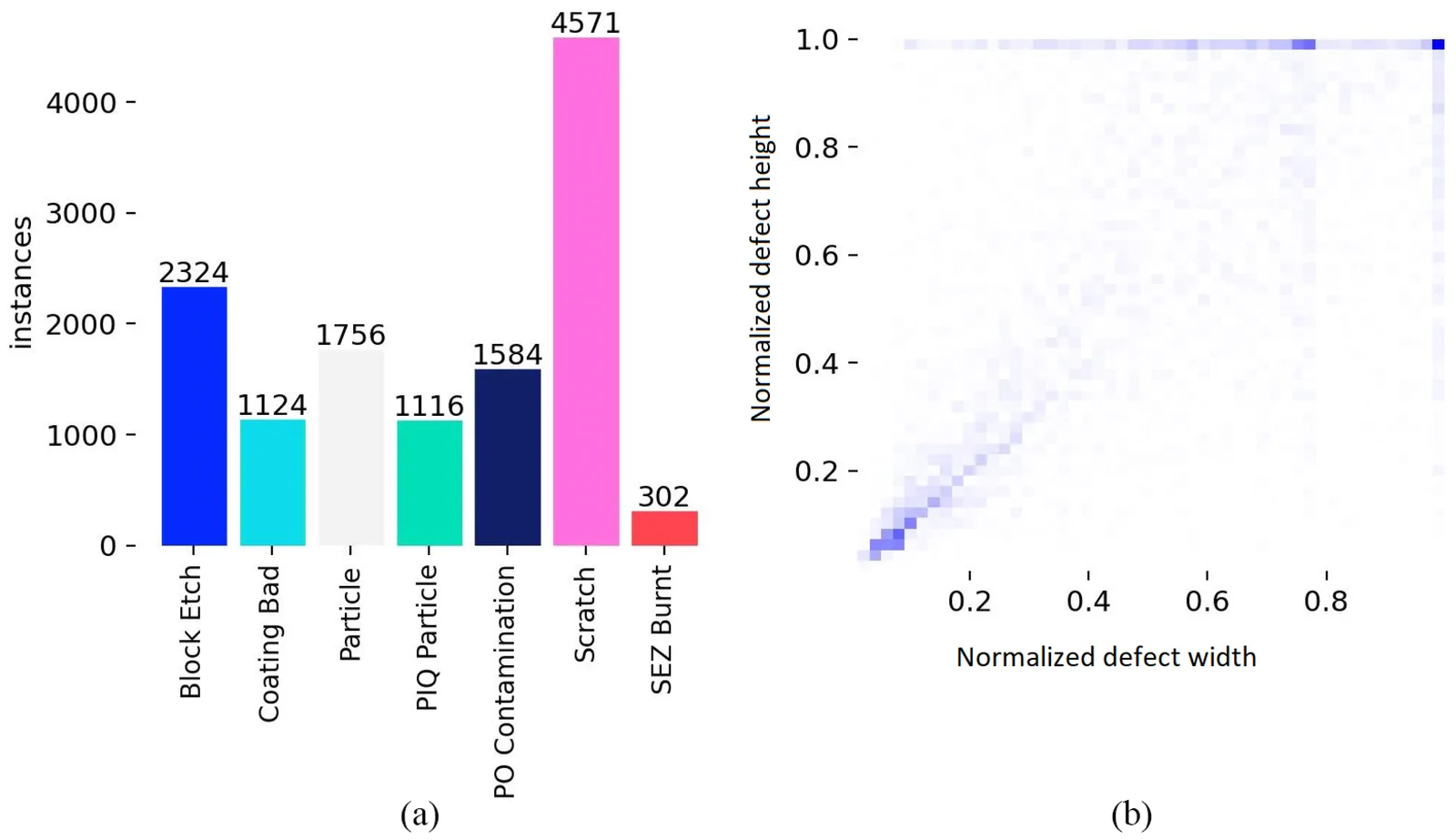
Label information: (**a**) distribution of each class, (**b**) distribution of normalized defect dimensions.

After data augmentation, the training set contains 12,777 samples, and the distribution of seven classes of defects is illustrated in Figure 2a. It can be found that the number of samples per class is highly imbalanced. For example, the ‘scratch’ category contains as many as 4571 instances, whereas the ‘SEZ burnt’ category includes only 302 instances. To mitigate this imbalance, the Slide loss was adopted by introducing an adaptive IoU-based threshold to differentiate easy and hard samples during training. Furthermore, the normalized ratios of label sizes and image sizes are presented in Figure 2b. The horizontal axis represents the ratio of the defect width to the full image width, and the vertical axis represents the ratio of the defect height to the full image height. It shows that both small and large defects coexist in the dataset, resulting in significant scale variation among defect instances.

It should be noted that the dataset used in this study is a publicly available industrial dataset for patterned wafer defect detection. Due to process confidentiality requirements, the precise optical metrology parameters of the original imaging system (e.g., objective magnification and calibrated pixel-to-physical size ratio) are not provided.

For dimensional reference, we estimated the physical scale of the images based on typical on-wafer interconnect geometries and standard configurations of industrial AOI systems. The equivalent pixel resolution is approximately 250–500 nm per pixel, which is consistent with common optical inspection settings in semiconductor manufacturing. Accordingly, the defect sizes represented in the dataset are estimated to range from sub-micron to several tens of microns, covering the typical defect scale encountered in semiconductor fabrication. It should be emphasized that this estimation is provided only for scenario description, rather than for precise metrological calibration.

### 3.2. Proposed PWDD-Net

**Figure 3:**
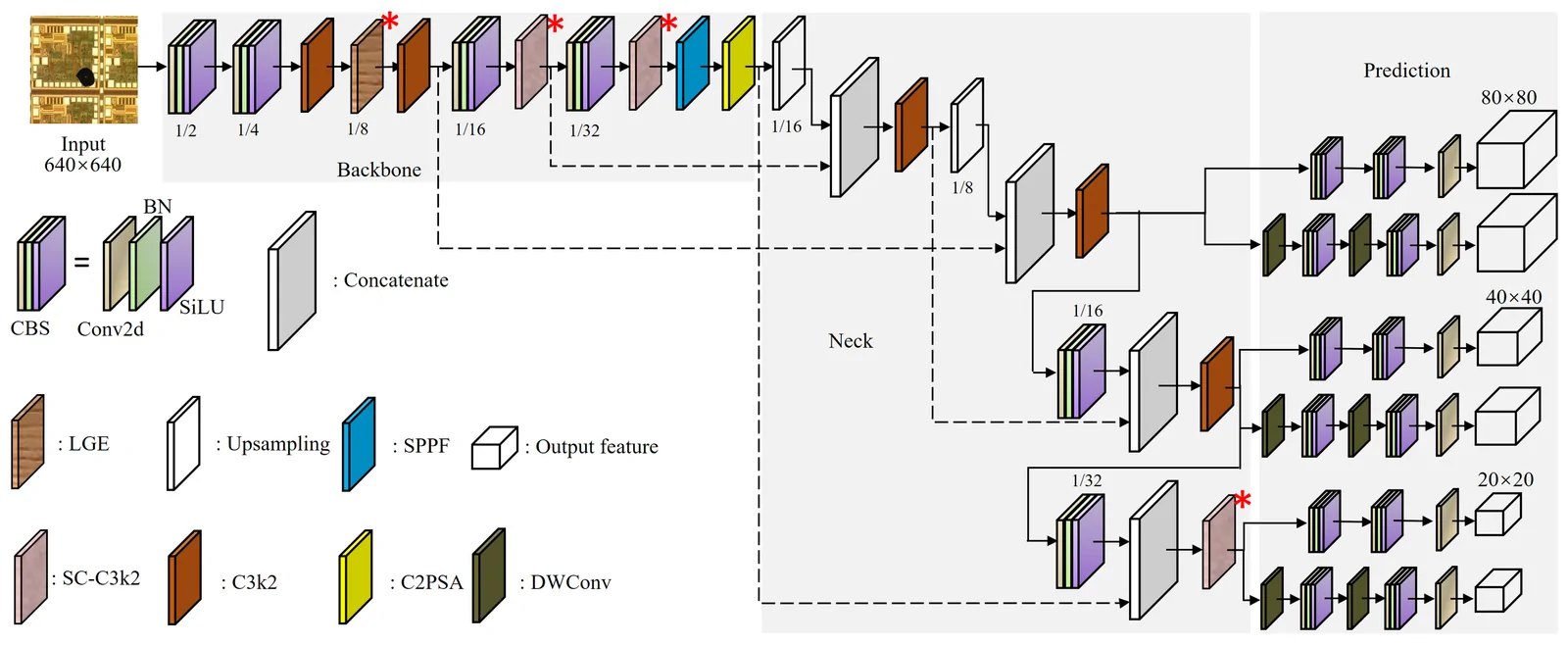
The architecture of propsoed PWDD-Net (Note: the marks * represent where the improvements were implemented).

The overall architecture of the proposed model is exhibited in Figure 3. Similar to YOLO11, the proposed PWDD-Net mainly comprises four components: the Input module, Backbone, Neck, and Prediction Head. The Input module is responsible for preprocessing input images, including adaptive resizing to a specific dimension during inference. In addition, optional built-in data augmentation strategies are provided and can be activated during training to enhance data diversity and improve model generalization. The backbone consists of standard convolutional layers, the proposed LGE block, C3k2 modules, the proposed SC-C3k2 modules, the spatial pyramid pooling-fast (SPPF) block and a C2PSA module. The main function is to downsample the input features and generate multi-scale feature maps with different channel dimensions. The SPPF block is employed to enlarge the receptive field and strengthen feature representation. The C2PSA module is adopted to emphasize critical features. The neck component contains a path aggregation network (PANet) [39] for multi-scale feature fusion. The prediction head outputs bounding boxes based on three multi-scale feature maps from neck part.

#### 3.2.1. Input

Concerning the input component, PWDD-Net adopts the same image preprocessing operations as YOLO11. Data augmentation techniques including HSV colour space transformation, translation, scaling and flipping are incorporated to expand the training images. During training, input images undergo uniform resizing and padding to ensure a standardized input resolution of 640 × 640 pixels. During inference, input images are adaptively resized with aspect-ratio preservation and minimal zero-padding, thereby reducing computational load and inference time.

#### 3.2.2. Backbone

Since it serves as the core feature extractor, the backbone is vital to enabling high detection accuracy in deep learning frameworks. The proposed PWDD-Net employs a redesigned backbone composed of standard convolutional layers, the proposed LGE blocks, C3k2 modules, the newly developed SC-C3k2 modules, an SPPF block and a C2PSA module. Among these components, the C3k2 modules, SPPF block and C2PSA module remain consistent with those in YOLO11.

(a) LGE block

Traditional convolution is effective at extracting local features but still remains limited in capturing global information. For example, edge information and corner information are particularly prone to being lost during downsampling process. Such local fine-grained details are vital for patterned wafer surface defect detection, as they encode context relationships within regions of interest (ROIs) and help distinguish weak defects from complex circuit backgrounds. In addition, conventional convolutional layers often involve a large number of parameters and high computational complexity.

**Figure 4:**
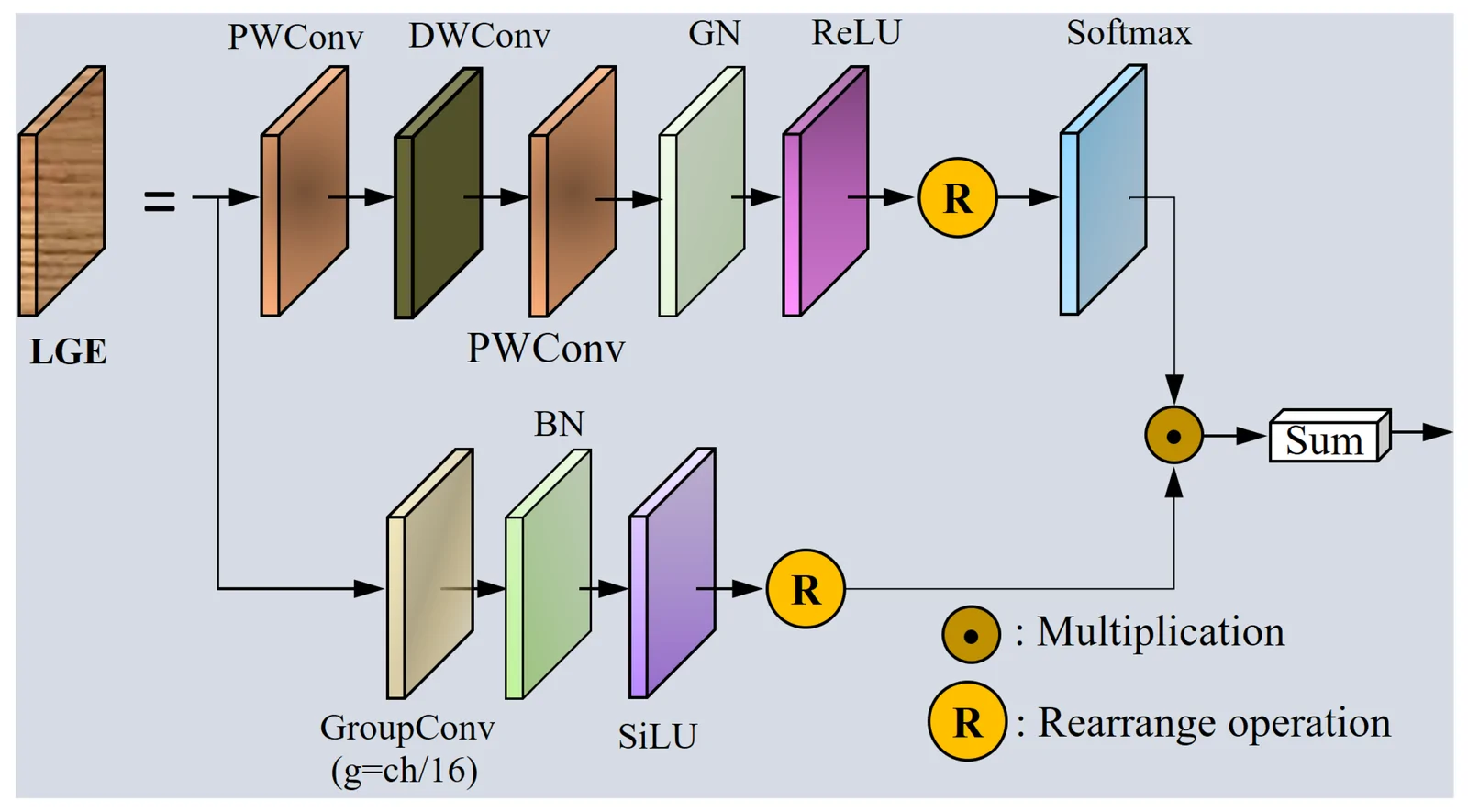
The structure of proposed LGE block.

To address these limitations, a lightweight gated extraction (LGE) block is introduced to enhance context feature representation while maintaining fine-grained information during feature transformation. The architecture of LGE block is illustrated in Figure 4. The proposed LGE block is designed to achieve efficient multi-scale feature extraction with limited parameter count. It consists of two parallel branches: a lightweight extraction branch for efficient feature representation and a gated regulation branch for adaptive feature extraction. Given an input feature map $x\in R^{B\times C\times H\times W}$, the LGE block can be formulated as

(1) $$y_{1}=PWConv(DWConv(PWConv(x))),$$

(2) $$y_{1}=Rea(ReLU\left(GN\left(y_{1}\right)\right)),$$

(3) $$y_{gc}=Softmax(y_{1}),$$

(4) $$y_{2}=SiLU(BN(GConv(x))),$$

(5) $$y_{c}=Rea(y_{2}),$$

(6) $$y_{o}=Sum(y_{c}*y_{gc},dim=-1).$$

where PWConv represents pointwise convolution, DWConv denotes depthwise convolution, GConv indicates group convolution, GN is group normalization, BN is batch normalization and Rea represents the rearrangement operation.

Inspired by LAE paper [40], the lightweight extraction branch aims to concentrate spatial information into the channel dimension while performing downsampling. First, group convolution is employed to map input features to the desired output dimensions. Group convolution with *g* groups reduces the parameter count to 1/g of standard convolution. Then, spatial information is reorganized through a rearrangement operation, transforming the feature map from a four-dimensional tensor ($y_{2}\in R^{B\times C\times H\times W}$) to a five-dimensional tensor ($y_{c}\in R^{B\times C^{\prime }\times H/2\times W/2\times n}$), where n=4 denotes the scaling factor. Here, a two-fold downsampling is performed to reduce both height and width dimensions by a factor of 2.

Similar to gated convolution [41], the gated regulation branch introduces a dynamic information modulation mechanism. Thus, the network can adaptively enhance informative features while suppressing irrelevant features. This branch employs two pointwise convolution layers [42], a 3 × 3 depthwise convolution [19] and a rearrangement operation. Subsequently, a softmax function is applied to generate gating coefficients ($y_{gc}$) within the range of 0 and 1, thereby regulating the representation of candidate features ($y_{c}$). Owing to the rearrangement operation, the output is also reshaped into a five-dimensional tensor, ensuring alignment with the lightweight extraction branch.

Then, the gating coefficients are multiplied with the rearranged features from the lightweight extraction branch, followed by summation along the scaling axis. The final output feature map $y_{o}$ has the size of (B, C, H/2, W/2).

The LGE module achieves efficient downsampling by integrating spatial and channel transformation with adaptive gated regulation. It preserves informative details while maintaining a lightweight architecture, enabling it suitable for patterned wafer defect detection tasks.

(b) SC-C3k2 module

Convolutional neural networks generally rely on local convolution kernels for feature extraction in object detection tasks. However, these convolutions have limited receptive fields and struggle to effectively map global spatial dependencies. Although stacking multiple convolutional layers can enlarge the receptive field, the modelling of global contextual information remains limited. To enhance feature representation while maintaining computational efficiency, self-calibrated convolution (SC-Conv) [25] is integrated into the C3k2 module of YOLO11. SC-Conv adopts a self-calibration mechanism which incorporates local pixel representations with wider contextual information. As a result, the model’s capability of capturing both spatial and semantic features is improved.

**Figure 5:**
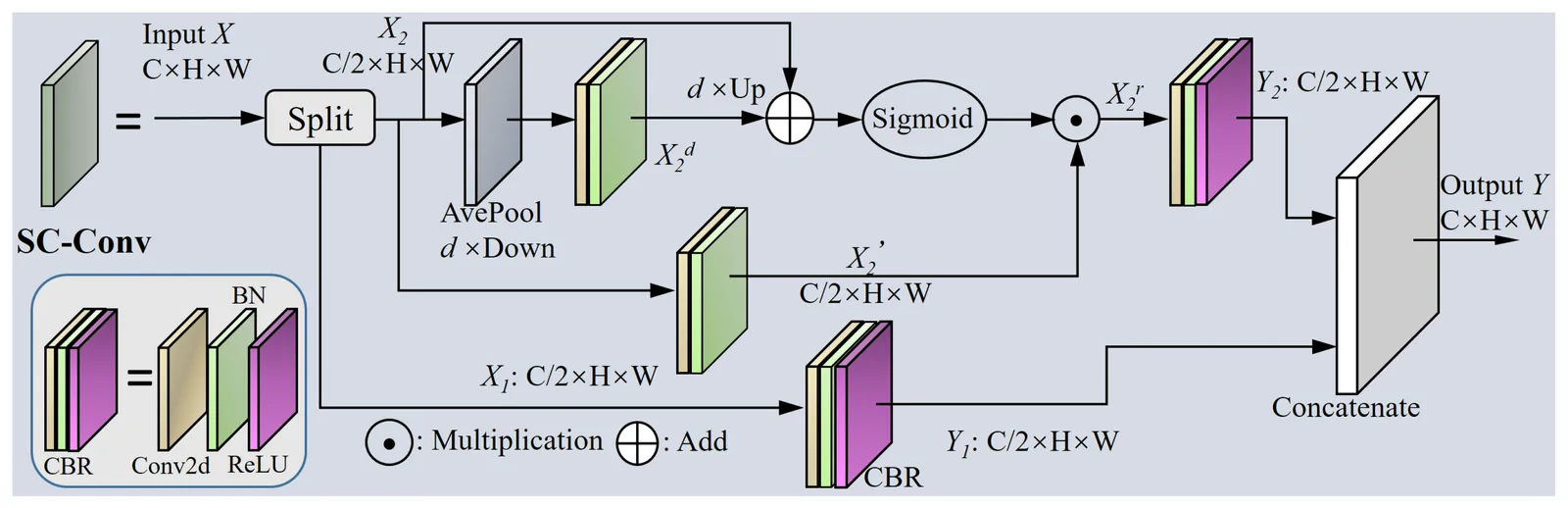
The structure of SC-Conv block.

The architecture of SC-Conv is illustrated in Figure 5. Given an input feature map *X*, it is first split into two groups along the channel dimension, denoted as $X_{1}$ and $X_{2}$. The $X_{1}$ branch undergoes a 3 × 3 standard convolution to extract local features, producing $Y_{1}$. Meanwhile, the $X_{2}$ branch is processed through two parallel paths. In the first path, $X_{2}$ is downsampled via average pooling followed by a 3 × 3 convolution to obtain $X_{2}^{d}$, thereby enlarging the receptive field. In the second path, $X_{2}$ passes through a 3 × 3 convolution to extract local features $X_{2}^{\prime }$.

The downsampled feature map $X_{2}^{d}$ is then upsampled and added to the identity mapping of $X_{2}$. The resulting feature is passed through a sigmoid function to generate calibration weights, which are subsequently applied to $X_{2}^{\prime }$ via element-wise multiplication, yielding the recalibrated feature $X_{2}^{r}$. A 3 × 3 convolution is then applied to $X_{2}^{r}$ to produce $Y_{2}$. Finally, $Y_{1}$ and $Y_{2}$ are concatenated along the channel dimension to obtain the output feature map *Y*. This strategy incorporates each local position with global context information to improve representation capability.

**Figure 6:**
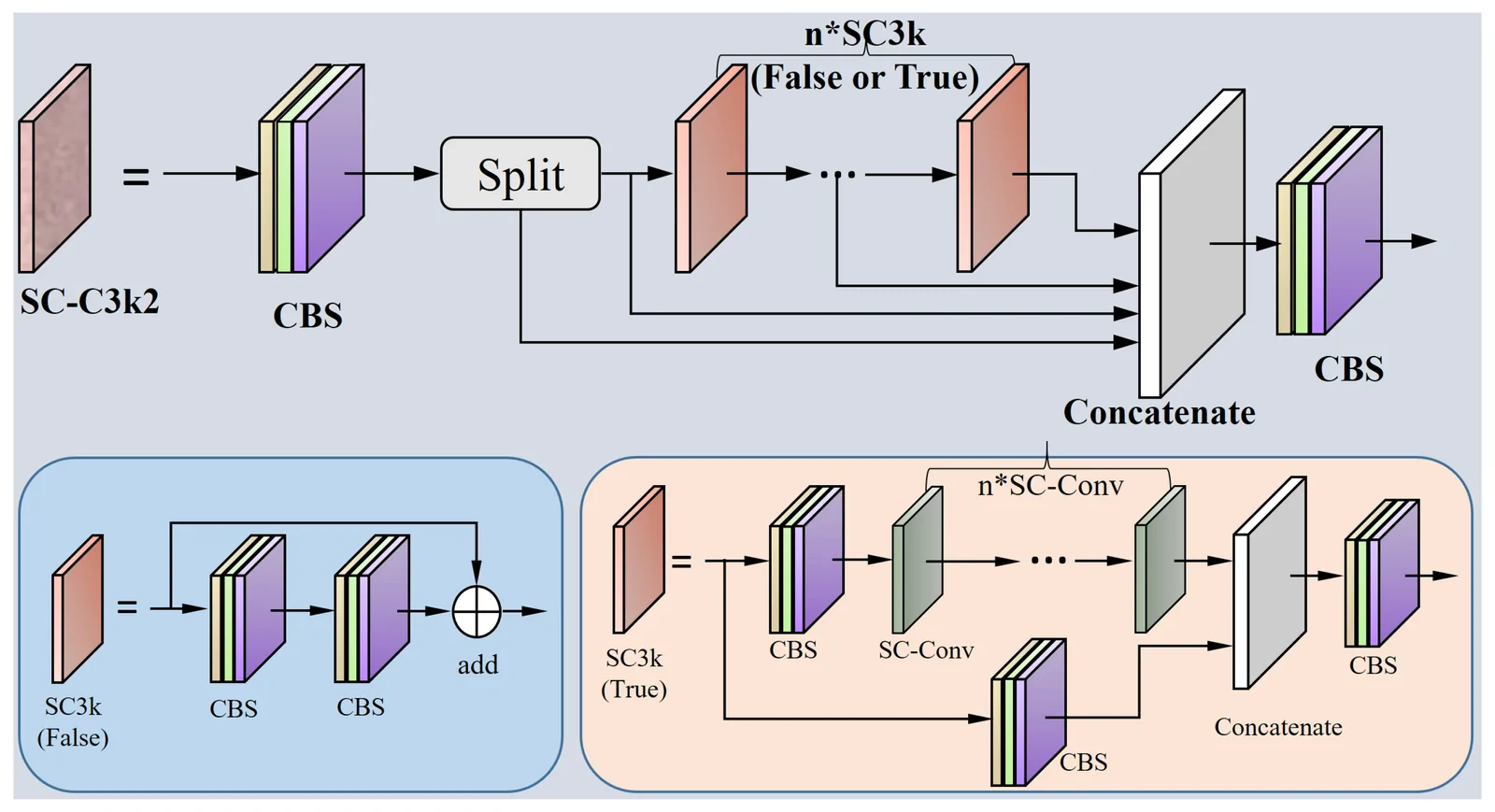
Architecture of the proposed SC-C3k2 module.

The C3k2 module is a fundamental feature extraction block in YOLO11. However, its core components are primarily built upon standard convolutional operations, which focus on local spatial information. In this study, the SC-Conv block is embedded into the C3k2 module to replace the original C3k block, forming the proposed SC-C3k2 module. The architecture of SC-C3k2 module can be found in Figure 6. The integration of SC-Conv enhances global feature representation while maintaining the lightweight design of the original structure. This modification strengthens discriminative feature extraction and improves detection performance. Consequently, the large scale variance issue among patterned wafer surface defects under complex circuit backgrounds, can be effectively mitigated.

#### 3.2.3. Neck

The neck structure of PWDD-Net is consistent with that of YOLO11 and adopts PANet [39] for multi-scale feature fusion. By integrating both top-down and bottom-up pathways, PANet effectively combines high-level semantic representations with low-level spatial information. Its bidirectional fusion strategy facilitates richer context information while preserving fine-grained localization details. Through cross-scale feature fusion, the neck part improves the defect representation under varying defect sizes and complex patterned backgrounds. As a result, the overall model becomes more robust to large-scale variation among wafer surface defects.

#### 3.2.4. Prediction Head

Several works have shown that decoupling classification and localization in detection heads can facilitate model optimization and improve prediction accuracy [43]. Therefore, PWDD-Net employs three independent decoupled detection heads, consistent with those in YOLO11. There are two branches in a single prediction head. In the localization branch, two 3 × 3 convolutions layers followed by a 1 × 1 convolution are implemented to refine bounding box regression. In contrast, the classification branch incorporates two lightweight depthwise convolutions to boost computational efficiency. Furthermore, larger convolution kernels are replaced with 1 × 1 convolutions. This architecture modification reduces parameter count and computation cost without compressing representation ability for accurate defect classification.

The overall optimization loss of PWDD-Net consists of a classification term and a bounding box regression term. For localization, the original regression loss of YOLO11 is preserved, integrating Complete IoU (CIoU) [44] with Distribution Focal Loss (DFL) to ensure precise bounding box regression. However, the classification branch is redesigned by integrating the conventional binary cross-entropy (BCE) loss with Slide Loss [45]. While class-weighted BCE addresses imbalance issue at the class level, it does not specifically account for the varying difficulty of individual instances. The Slide Loss function can be formulated as Equation (7), where μ is the average value of the IoU of all bounding boxes.

(7) $$f\left(x\right)=\left\{\begin{array}{c} 1,IoU\leq \mu -0.1\\ e^{1-\mu },\mu -0.1<IoU<\mu \\ e^{1-IOU},IoU\geq \mu \end{array}\right.$$

Samples with IoU values lower than μ are regarded as negative samples, while those with IoU values higher than μ are regarded as positive samples. However, instances near the decision boundary (i.e., IoU close to μ) are always difficult to classify and tend to produce large losses. To better utilize these hard instances, a dynamic weighting function, termed Slide, is introduced to assign higher weights to these boundary samples, thereby increasing their contribution during training. Consequently, Slide Loss can dynamically reweight the classification loss based on the IoU between prediction and ground-truth boxes. This adaptive reweighting mechanism mitigates the bias toward majority wafer defect classes during training. Thus, minority defect categories receive greater attention, enhancing model robustness and promoting detection performance.

## 4. Results and Discussion

### 4.1. Experimental Configuration

A custom-built computer equipped with an Intel(R) Xeon(R) Silver 4214R CPU, 64 GB DDR4 memory, an NVIDIA GeForce RTX 3080Ti GPU (12 GB GDDR6X video memory) and a 1 TB solid-state drive was used to conduct all the experiments. The environment was based on PyTorch 2.5.1, Python 3.12, CUDA 12.4 under the Ubuntu 22.04 operating system. The models were trained without pre-trained weights. The batch size was set to 16, and the input image was fixed at 640 × 640 × 3. The AdamW optimizer was adopted with an initial learning rate of 0.01. Each model was trained for 200 epochs.

### 4.2. Evaluation Metrics

In order to evaluate model performance, we apply the confusion matrix to calculate precision and recall, as shown in Table 2. Their definitions are formulated in Equation (8).

(8) $$\begin{array}{cc} Precision & =\frac{TP}{TP+FP}\\ Recall & =\frac{TP}{TP+FN} \end{array}$$

Because precision and recall exhibit a trade-off relationship, we adopt average precision (AP) to provide a more reasonable assessment of detection performance. AP is defined as the area under the precision–recall curve:

(9) $$AP=\int_{0}^{1}p\left(r\right)dr,$$

where *p* denotes precision and *r* represents recall.

By averaging the AP values of all defect categories, we obtain the mean average precision at an IoU threshold of 0.5 (mAP@0.5). Then, we calculate mAP@0.5:0.95 metric, defined as the mean of AP values at IoU thresholds ranging from 0.5 to 0.95 with an increment of 0.05.

### 4.3. Experiment Results

#### 4.3.1. Ablation Results

To evaluate the contributions of the proposed LGE block, SC-C3k2 module, and Slide loss, ablation experiments are performed. Based on the YOLO11-nano baseline, each proposed improvement is incorporated gradually. All the models are evaluated on the same test dataset. The ablation results are presented in Table 3. The proposed modules lead to robust detection gains over the baseline, demonstrating their effectiveness.

As indicated in Table 3, integrating the proposed LGE block to replace standard convolution leads to noticeable improvements in detection performance. Compared to the YOLO11-nano baseline, the modified model achieves increases of 2.4% in mAP@0.5 and 0.5% in mAP@0.5:0.95. In addition, the precision rises by 3.8%, reaching 67.3%. Although the recall metric experiences a slight reduction of 1.4%, the overall performance improvement demonstrates the effectiveness of the LGE block. The enhanced performance introduced by the LGE block results from its excellent context representation ability and fine-grained feature extraction. Conventional convolutions generally capture local features and may lose edge information during downsampling. In contrast, the LGE block employs an adaptive gated mechanism and transforms spatial information into the channel dimension. This design enables more effective cross-location feature interaction and effective concentration on important defect features under complex circuits. Meanwhile, the lightweight group convolution strengthens representation capability without increasing computational cost. Thus, LGE enhances discriminative feature learning for small-scale and low-contrast defects, resulting in improved detection accuracy and efficient inference speed.

Then, we replace the original C3k2 modules with the proposed SC-C3k2 modules. Common convolutional operations mainly focus on local spatial features instead of capturing global spatial information. By integrating SC-Conv block into the C3k2 module, each local feature is recalibrated with wider context information. Moreover, the computation complexity is not increased. This modification enhances the interaction of spatial semantic information, strengthening defect representation. As a result, the feature discrimination for varying defect scales is improved. The large scale variation issue in patterned wafer surface defect detection can be effectively mitigated. As presented in Table 3, incorporating SC-C3k2 leads to consistent performance improvements. The precision is increased by 3.9% to 71.2%, while recall is boosted by 0.7% to 72.6%. Moreover, mAP@0.5 and mAP@0.5:0.95 are increased by 0.9% and 0.4%, respectively, further validating the effectiveness of the proposed module.

Although the implementation of the Slide loss leads to a slight reduction in precision, the recall metric is increased by 1.0% to 73.6%. Furthermore, mAP@0.5 value is increased by 0.9% to 74.4%, and mAP@0.5:0.95 is improved by 1.2% to 46.5%. The Slide loss reshapes the classification task by reweighting samples according to their IoU values. This dynamic weighting mechanism enhances the representation of minority defect categories, contributing to the apparent improvements in recall and mAP@0.5:0.95. The overall detection performance under class imbalance is effectively strengthened.

**Figure 7:**
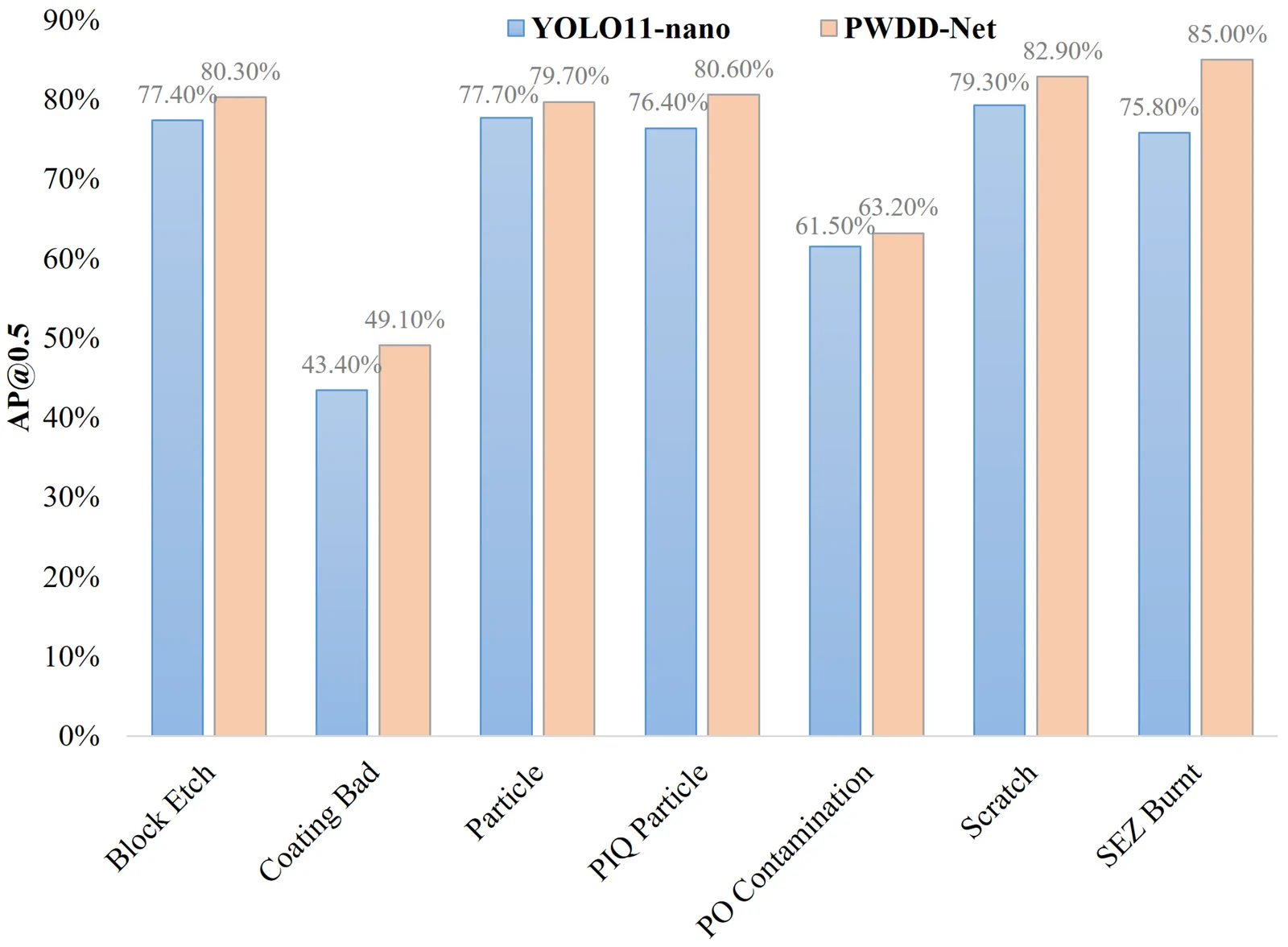
Class-wise detection performance of PWDD-Net and YOLO11-nano.

We compare the class-wise detection performance of PWDD-Net and the original YOLO11-nano, as shown in Figure 7. The detection metrics for all the seven classes are increased. The increments are 2.9% (i.e., 80.3–77.4%) for ‘Block Etch’, 5.7% (i.e., 49.1–43.4%) for ‘Coating Bad’, 2.0% (i.e., 79.7–77.7%) for ‘Particle’, 4.2% (i.e., 80.6–76.4%) for ‘PIQ Particle’, 1.7% (i.e., 63.2–61.5%) for ‘PO Contamination’, 3.6% (i.e., 82.9–79.3%) for ‘Scratch’ and 9.2% (i.e., 85.0–75.8%) for ‘SEZ Burnt’. The results suggest that the class imbalance issue has been effectively mitigated.

In summary, the proposed PWDD-Net demonstrates superior performance compared with YOLO11-nano in patterned wafer surface defect detection. It attains a precision of 70.3% and a recall of 73.6%, corresponding to increases of 6.8% and 0.3% over the baseline, respectively. Moreover, the model achieves mAP@0.5 of 74.4% and mAP@0.5:0.95 of 46.5%, yielding improvements of 4.2% and 2.1%. Notably, the inference speed remains at 78 FPS, demonstrating that the performance gains are achieved without sacrificing detection efficiency.

**Figure 8:**
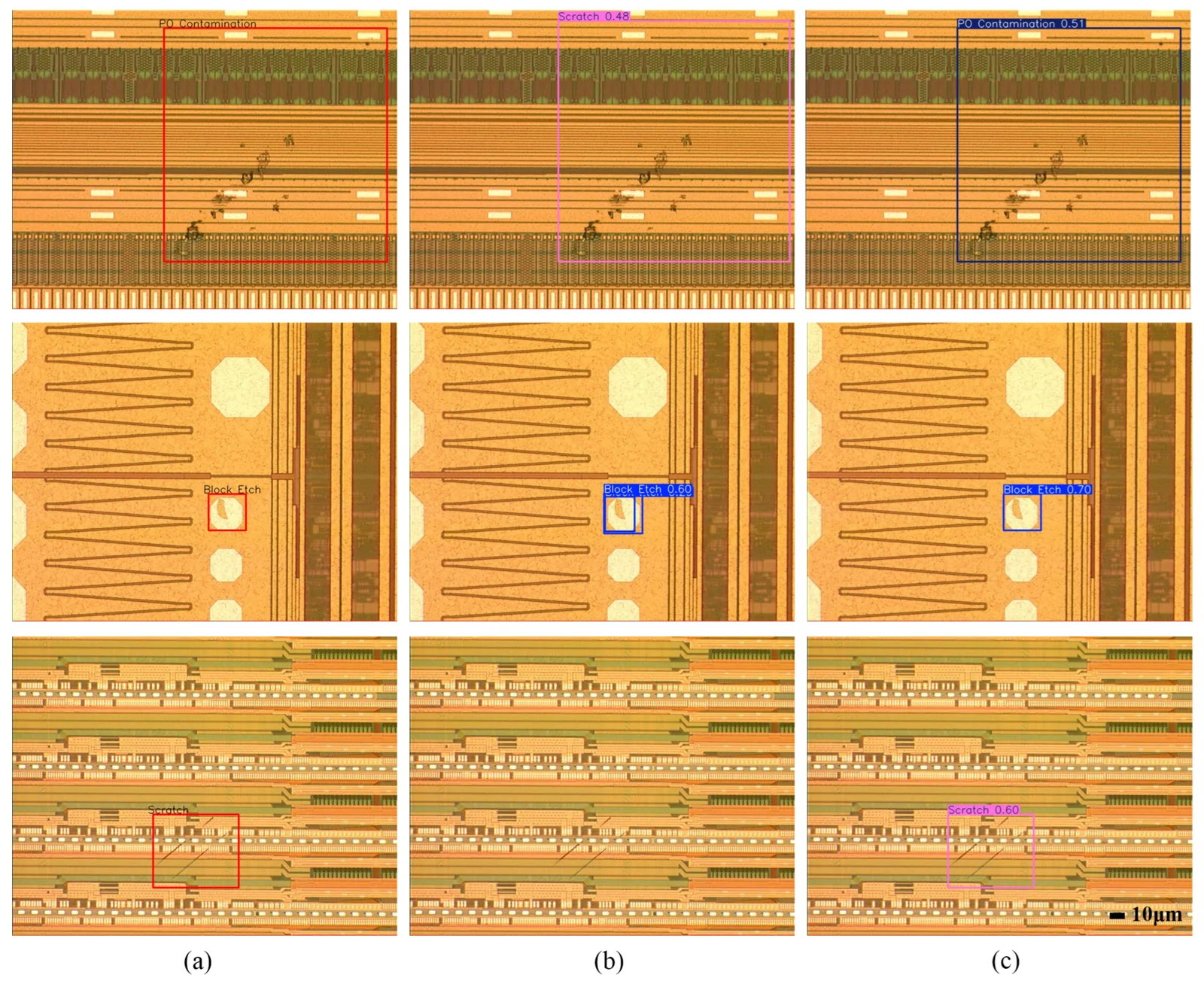
Representative detection examples by different models: (**a**) Ground truth. (**b**) YOLO11-nano. (**c**) PWDD-Net. An estimated 10 μm scale bar is only used to indicate the approximate physical scale of the defects.

Figure 8 presents some representative detection examples to further evaluate model performance. The baseline YOLO11-nano exhibits several failed detections, including identifying ‘PO Contamination’ to ‘Scratch’, generating redundant bounding boxes for the ‘Block Etch’ class, and missing ‘Scratch’ instances in some cases. By contrast, PWDD-Net effectively avoids these issues and provides accurate predictions for defects with different scales. These visual results confirm the effectiveness of the proposed enhancements.

#### 4.3.2. Comparison Results

The proposed PWDD-Net was compared with several state-of-the-art object detection frameworks, including RetinaNet [46], EfficientDet [47], RT-DETRv2s [48], YOLOv8-nano [49], YOLOv8-small [49], YOLOv9-tiny [50], YOLOv10-nano [51], YOLO11-small [23], YOLO12-nano [52] and YOLO26-nano [53]. The comparative experiment was conducted in terms of model parameter count, detection mAP, and inference speed.

As shown in Table 4, PWDD-Net achieves superior detection accuracy compared with several representative baselines. It attains 74.4% mAP@0.5 and 46.5% mAP@0.5:0.95. However, RetinaNet, EfficientDet and RT-DETRv2 exhibit lower metrics on the wafer defect dataset. The YOLO series networks provide a favourable balance between detection speed and accuracy, with all tested variants demonstrating competitive results. Among them, YOLOv8-nano reaches the highest inference speed at 91 FPS. YOLOv9-tiny maintains the smallest parameter count and exhibits competitive detection results, although it suffers from relatively higher latency. Different from them, PWDD-Net attains the best mAP metrics while retaining a lightweight design. Its inference speed remains at 78 FPS, demonstrating a favorable balance between accuracy and efficiency.

**Figure 9:**
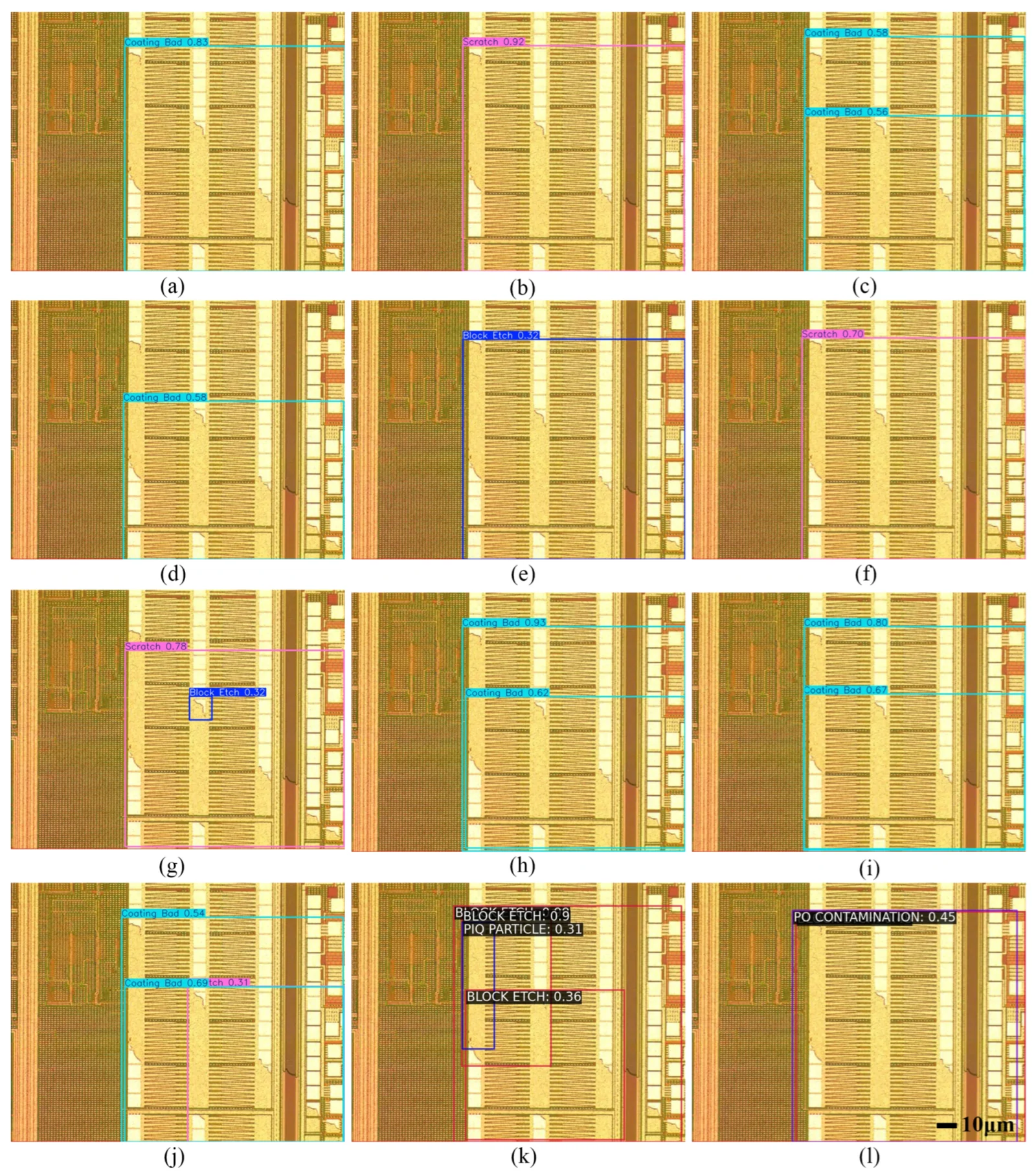
Detection examples of wafer surface defects by different models: (**a**) PWDD-Net, (**b**) YOLO26-nano, (**c**) YOLO12-nano, (**d**) YOLO11-small, (**e**) YOLO11-nano, (**f**) YOLOv10-nano, (**g**) YOLOv9-tiny, (**h**) RT-DETRv2s, (**i**) YOLOv8-small, (**j**) YOLOv8-nano, (**k**) EfficientDet, (**l**) RetinaNet. An estimated 10 μm scale bar is provided for dimensional reference.

Patterned wafer surface defect detection results obtained by the proposed model and other advanced detectors are illustrated in Figure 9. EfficientDet and RetinaNet exhibit clear misclassification errors, detecting the ‘Coating Bad’ sample as other defect categories, such as ‘PO Contamination’, ‘Block Etch’ and ‘PIQ Particle’. RT-DETRv2s, although capable of detecting the target, generates redundant bounding boxes for the same instance. The evaluated YOLO models also show a variety of prediction errors. For instance, YOLO26-nano, YOLOv10-nano, YOLOv9-tiny and YOLO11-nano misidentify the ‘Coating Bad’ defect as either ‘Scratch’ or ‘Block Etch’. YOLOv9-tiny additionally generates incorrect duplicate detections. Similarly, both of YOLOv8-small and YOLO12-nano produced redundant bounding boxes. Although YOLOv8-nano correctly recognizes the ‘Coating Bad’ sample, it still introduces redundant and false positive predictions.

In contrast, PWDD-Net demonstrates more accurate localization and classification results with minimal negative outputs. The integration of the LGE block, SC-C3k2 module, and Slide loss contributes to improved feature discrimination and balanced classification. However, due to the large scale variance and strong interference from complex circuit textures, all evaluated models including PWDD-Net, experience occasional missed detections for extremely small defects.

## 5. Conclusions

A novel PWDD-Net framework is developed for patterned wafer surface defect detection, incorporating a few enhancements. First, the LGE block, integrating group convolution and a gated modulation mechanism, is designed to strengthen fine-grained feature extraction and enhance discriminative representation. Thus, the LGE block enables adaptive capture of richer contextual information and high-resolution details. Second, through embedding a self-calibration block, a lightweight SC-C3k2 module is newly proposed to improve the global feature representation capability without introducing extra parameters. Finally, the Slide loss is employed to mitigate the class imbalance issue, enabling more stable optimization and improving classification accuracy.

The effectiveness of PWDD-Net is validated through several experiments, where the model demonstrates superior detection performance for seven classes of wafer defects. It attains a mAP@0.5 of 74.4% and a mAP@0.5:0.95 of 46.5%, respectively. Notably, these performance increments are achieved without increasing the parameter count, allowing PWDD-Net to maintain an inference speed of 78 FPS. Owing to its strong fine-grained feature extraction capability and lightweight design, the proposed PWDD-Net also exhibits the potential for nanoscale wafer defect characterization. Thus, it provides an efficient and intelligent detection solution for quality control in micro- and nanoscale manufacturing.

This study focuses on seven categories of patterned wafer surface defects. However, semiconductor manufacturing produces a wide range of defect types. Therefore, future research will expand the dataset to include additional defect classes. Moreover, further extension and optimization of the proposed detection framework to accommodate finer nanoscale defect scenarios in advanced semiconductor process nodes will constitute an important research direction. Although the proposed model demonstrates promising performance, class imbalance issue still exists. Future work will further develop more specialized loss functions to address imbalanced data distributions. Besides, advanced generative adversarial networks and diffusion models could be employed to generate a mass of defects, thereby enhancing dataset diversity and model generalization. The current detection accuracy still needs improvement. Future research will explore more effective feature representation mechanisms and optimization strategies to further enhance detection precision.

## Figures and Tables

**Table 1:** The number and input size of images in the subsets.

Subset	Training	Validation	Test
Number	12,684	906	454
Input size	640 × 640 × 3	640 × 640 × 3	640 × 640 × 3

**Table 2:** Confusion matrix.

	Prediction	True	False
Ground Truth			
True		True positive (TP)	False negative (FN)
False		False positive (FP)	True negative (TN)

**Table 3:** Ablation experimental results.

Models	Precision (%)	Recall (%)	mAP@0.5 (%)	mAP@0.5:0.95 (%)	FPS
YOLO11-nano	63.5	73.3	70.2	44.4	78
+LGE	67.3	71.9	72.6	44.9	77
+SC-C3k2	71.2	72.6	73.5	45.3	78
+Slide loss (PWDD-Net)	70.3	73.6	74.4	46.5	78

**Table 4:** Comparison experiment results.

Models	Parameters (M)	mAP@0.5 (%)	mAP@0.5:0.95 (%)	FPS
RetinaNet	37.74	63.1	42.5	17
EfficientDet-d0	3.92	62.5	36.5	58
YOLOv8-nano	3.20	66.6	44.4	91
YOLOv8-small	11.14	67.4	46.0	72
RT-DETRv2s	20.09	61.5	42.4	26
YOLOv9-tiny	2.01	68.4	45.9	55
YOLOv10-nano	2.71	65.7	43.3	85
YOLO11-nano	2.60	70.2	44.4	78
YOLO11-small	9.43	66.2	43.1	63
YOLO12-nano	2.57	69.0	46.0	66
YOLO26-nano	2.51	69.1	43.4	86
PWDD-Net	2.59	74.4	46.5	78

## Data Availability

The raw data supporting the conclusions of this article will be made available by the authors on request.
